# Strains of the *Propionibacterium acnes* type III lineage are associated with the skin condition progressive macular hypomelanosis

**DOI:** 10.1038/srep31968

**Published:** 2016-08-24

**Authors:** Emma Barnard, Jared Liu, Eliza Yankova, Silvana M. Cavalcanti, Marcelo Magalhães, Huiying Li, Sheila Patrick, Andrew McDowell

**Affiliations:** 1 Centre for Infection & Immunity, School of Medicine, Dentistry & Biomedical Sciences, Queen’s University, Belfast, UK; 2 Department of Molecular and Medical Pharmacology, Crump Institute for Molecular Imaging, David Geffen School of Medicine, UCLA, Los Angeles, California, USA; 3 Northern Ireland Centre for Stratified Medicine, Biomedical Sciences Research Institute, C-TRIC Building, Altnagelvin Area Hospital, University of Ulster, Londonderry, UK; 4 Department of Dermatology, University of Pernambuco, Recife, Brazil; 5 Department of Microbiology, Federal University of Pernambuco, Recife, Brazil; 6 UCLA-DOE Institute for Genomics and Proteomics, Los Angeles, California, USA

## Abstract

Progressive macular hypomelanosis (PMH) is a common skin disorder that causes hypopigmentation in a variety of skin types. Although the underlying aetiology of this condition is unclear, there is circumstantial evidence that links the skin bacterium *Propionibacterium acnes* to the condition. We now describe the first detailed population genetic analysis of *P. acnes* isolates recovered from paired lesional and non-lesional skin of PMH patients. Our results demonstrate a strong statistical association between strains from the type III phylogenetic lineage and PMH lesions (*P* = 0.0019), but not those representing other phylogroups, including those associated with acne (type IA_1_). We also demonstrate, based on *in silico* 16S rDNA analysis, that PMH isolates previously recovered from patients in Europe are also consistent with the type III lineage. Using comparative genome analysis, we identified multiple genomic regions that are specific for, or absent from, type III strains compared to other phylogroups. In the former case, these include open reading frames with putative functions in metabolism, transport and transcriptional regulation, as well as predicted proteins of unknown function. Further study of these genomic elements, along with transcriptional and functional analyses, may help to explain why type III strains are associated with PMH.

The common and worldwide skin disorder progressive macular hypomelanosis (PMH) is characterised by asymptomatic, non-scaly hypochromic macules found mainly on the front and back of the trunk, and is often mistaken for pityriasis versicolor and pityriasis alba; the condition rarely affects the face. While PMH can affect both sexes, it is much more common in young females and can spontaneously disappear after mid-life. Histologically, PMH lesions have a normal looking dermis but diminished pigment in the epidermis, with small aggregated and membrane-bound melanosomes replacing the large melanosomes found in normal skin^1^. While the underlying cause of this condition remains unclear, there is growing evidence to suggest that the Gram-positive anaerobic skin bacterium *Propionibacterium acnes* may play an important role in the aetiology of the condition^2^,^3^,^4^. In particular, *P. acnes* is frequently cultivated from lesional biopsies and has been observed in histological sections, but recovery from peri-lesional, normal-looking skin is infrequent^5^. Counts of the bacterium in lesional skin are also much higher as measured by quantitative real-time PCR (qPCR)^4^. Furthermore, analysis of lesions under a Wood’s lamp reveals a red follicular fluorescence due to the production of porphyrins by the bacterium, a phenomenon not observed with healthy pigmented skin on the trunk region^2^,^4^. Consistent with a role for *P. acnes* in PMH, the administration of topical and oral antibacterials and UVA phototherapy often proves an effective treatment leading to repigmentation of the lesions^5^,^6^,^7^,^8^.

A previous study by Relyveld *et al*. using a combination of Amplified Fragment Length Polymorphism (AFLP) analysis, 16S rDNA sequencing, and biochemical profiling, found that *P. acnes* isolates cultivated from the PMH biopsy tissue of patients in the Netherlands, classified as ‘DNA group 3’, were genetically and phenotypically distinct from isolates associated with acne vulgaris^3^. This provides initial evidence that a specific phylogenetic grouping of *P. acnes*, or possibly a novel *Propionibacterium* species, is associated with PMH^3^. To date, however, a detailed population genetic analysis of isolates recovered from PMH lesions has been lacking, and it remains unclear which, if any, of the now well described phylogroups of *P. acnes* (types IA_1_, IA_2_, IB and IC, II and III) may be associated with the condition. Such studies are important since specific lineages of the bacterium with an increased potential to cause infection may exist, while others may prove to be associated with health^9^,^10^,^11^,^12^,^13^; the latter may therefore provide the basis for the development of novel probiotic therapies to treat PMH if disease-causing lineages are identified. In this study, we describe the first detailed population genetic analysis of *P. acnes* strains isolated from paired lesional and non-lesional skin of patients with PMH. We also compare the genomes of a number of *P. acnes* type III strains, including those isolated from the skin of PMH patients, with *P. acnes* strains representing all other phylogenetic divisions of the bacterium.

## Results

### *P. acnes* type III lineage is significantly associated with PMH

**Figure 1:**
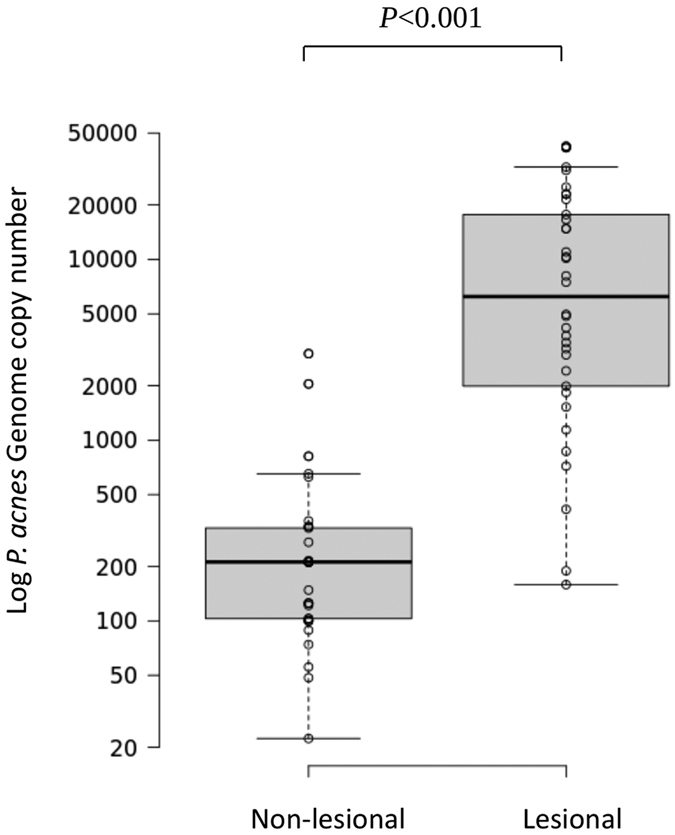
Boxplot of *P. acnes* genome copy number for paired non-lesional and lesional skin samples from patients with PMH. Centre lines reflect medians and box limits indicate the 25th and 75th percentiles.

Levels of *P. acnes* in the lesional skin of this study cohort were significantly higher than non-lesional skin samples based on genome copy number (*P* < 0.001 Wilcoxon Signed Rank test; Fig. 1). Consistent with these results, culture-based analysis revealed a significant association between the presence of *P. acnes* in the lesions of 27/34 (79%) patients with PMH versus adjacent and paired non-lesional, normal-looking skin samples which mostly showed no evidence of any growth (*P* < 0.0001 McNemar’s test) (OR = 27; 95% CI = 4.45–1105) (Table 1). Of the seven lesional skin samples that were classified as culture-negative, four did display some growth of *P. acnes* but, for stringency in the identification of culture-positive samples, they were classified as negative since their genome copy numbers were low and below our cut-off value. Multiplex PCR analysis of multiple *P. acnes* colonies revealed little evidence of mixed phylogroups in the lesions based on culture, except in two cases, which is consistent with previously published results^3^ (Table 1). Of the other lesional biopsy samples positive by culture (n = 25), 52% had the type III lineage present versus 16% with type IA_1_, 12% with type IA_2_, 4% with type IB and 16% with type II (Table 1). Upon Gram staining, all PMH isolates identified as type III had the characteristic elongated cellular morphology that we originally described for this genetic division^14^ (Supplementary Fig. S1). One patient had growth of *P. acnes* from adjacent non-lesional skin only; in this case, both type III and IA_1_ strains were recovered (Table 1). The differences in the number of type III strains isolated from the PMH lesions versus the paired non-lesional skin was statistically significant using the McNemar’s test for paired nominal data, thus demonstrating a striking association between this genetic division and PMH (*P* = 0.0019; OR = 14; 95% CI = 2.13–592). In contrast, no significant difference was found between paired lesion and non-lesional skin of the patients for the presence of types IA_1_, IA_2_, IB and II lineages (*P* > 0.05).

### AFLP DNA group 3 strains are also consistent with the *P. acnes* type III lineage

**Figure 2:**
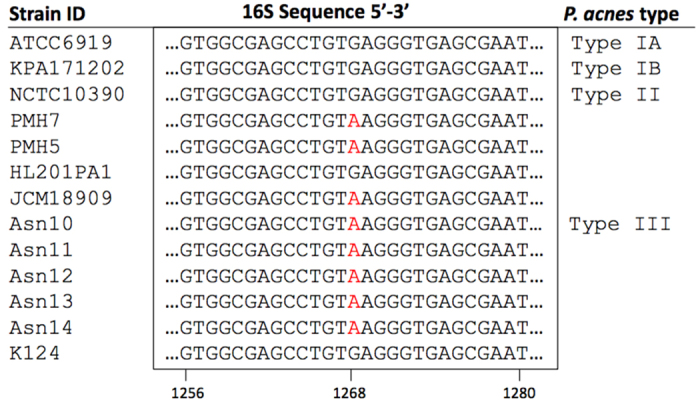
Alignment of the 16S rDNA sequence from ATCC6919 (type IA_1_), KPA171202 (type IB) and NCTC10390 (type II) versus type III isolates. The 16S rDNA G>A SNP described by Relyveld *et al*.^3^ as a genetic marker of AFLP DNA group 3 strains is highlighted. This SNP was present in eight of the type III isolates analysed, but absent in type strains from the other major *P. acnes* lineages.

Relyveld *et al*. previously reported that the AFLP DNA group 3 strains recovered from PMH lesions differed from the 16S rDNA gene sequence of the type IA_1_ strain ATCC6919 (NCTC737) (accession number AB042288.1) by a single nucleotide polymorphism (SNP), G1243A^3^. Alignment of the 16S rDNA sequences from ATCC6919, KPA171202 (type IB), NCTC10390 (type II) and 10 publically available type III isolates confirmed the presence of this specific SNP in eight of the type III isolates (80%), while two were identical to ATCC6919, KPA171202 and NCTC10390 at this site (Fig. 2). Inspection of the 16S rRNA gene sequences from 97 sequenced *P. acnes* genomes currently publicly available, and covering all other phylogroups (IA_1_, IA_2_, IB, IC and II), found no evidence of this SNP outside of the type III division. Therefore, this SNP would appear specific to strains from the type III lineage. Further inspection of published 16S metagenomic sequence data of *P. acnes* populations within the skin revealed that the 16S rRNA SNP of ribotype (RT) 9, G1268A (*Escherichia coli* equivalent coordinate; Supplementary Fig. S2) rid="b12"/>, corresponds to this type III-specific SNP, suggesting that RT9 may belong to type III.

### Biochemical analysis of type III isolates from PMH lesions

Analysis of the biochemical phenotype of three representative type III strains recovered from PMH lesions using the Rapid ID 32A multi-test identification system failed to correctly identify the isolates as *P. acnes* (Supplementary Table S1. In contrast, the acne type IA_1_ isolates ATCC6919 (NCTC737) and hdn-1, representing AFLP DNA group 1, and type II isolates NCTC10390 and BR-26, representing AFLP DNA group 2, were correctly identified as *P. acnes* with an identity of 99.9% (Supplementary Table S1). With the type III PMH strains tested, the current Rapid ID 32A database incorrectly identified them as *Clostridium tetani* (97.5% highest identity), *Eggerthella lenta* (65.3% highest identity) or *Fusobacterium nucleatum* (42.1% highest identity) species (Supplementary Table S1); members of these species are anaerobic bacilli and potentially pathogenic to humans.

### Multilocus Sequence Typing (MLST) of isolates from PMH lesional and non-lesional skin

To provide a more detailed picture of the population genetic structure of strains recovered from lesional and non-lesional skin, we selected a total of 24 isolates pre-screened by multiplex PCR for higher resolution MLST_8_ analysis. One strain was from non-lesional skin (type III) and 23 were from PMH lesions, the majority of which were type III (n = 15), along with two strains each of types IA_1_, IA_2_, IB and II (n = 8). MLST confirmed the phylogroup designations obtained by multiplex PCR analysis of the isolates, and further resolved a total of five distinct type III genotypes represented by ST75, ST76, ST77 and two new STs. All five STs were recovered from lesional sites, while ST76 also represented the type III isolate from non-lesional skin. Lesional isolates of types IA_1_, IA_2_ and IB phylogroups had very common, globally disseminated genotypes represented by ST1 (type IA_1_), ST2 (type IA_2_), ST22 (type IA_2_) and ST5 (type IB), while type II strains were represented by ST69 and a new ST. *In silico* analysis of recently published draft genome sequences of two type III isolates (PMH5 and PMH7) also recovered from the lower back of patients with PMH in Aalborg, Denmark (from skin swabs) revealed the genotype ST33^15^. All type III STs identified belonged to the previously described type III clonal complex (CC), CC77 which has ST77 as the ancestral or founding genotype^10^,^11^.

### Genomic characteristics of type III strains

**Figure 3:**
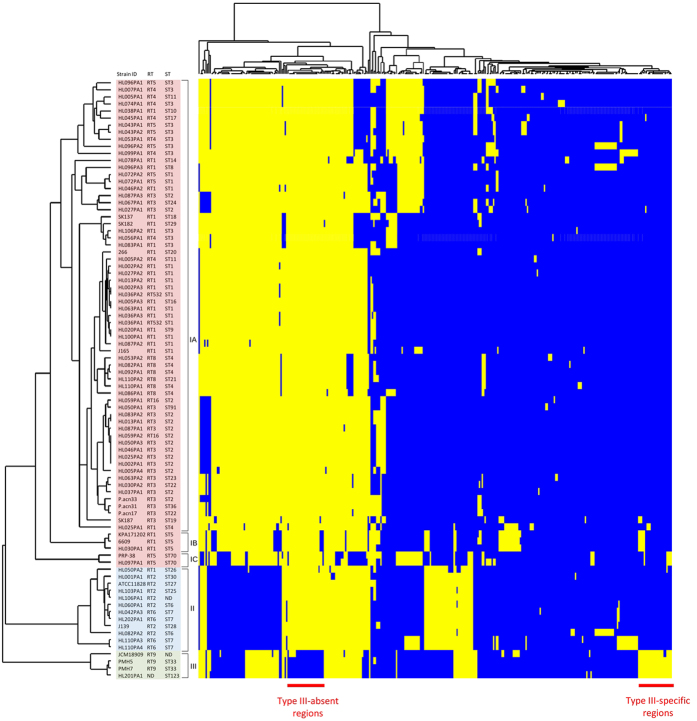
Presence and absence of non-core genomic regions across 85 *P. acnes* genomes. A total of 336 non-core regions (columns) from 85 *P. acnes* genomes (rows) are clustered based on similar patterns of presence (yellow) and absence (blue) of each region. Size and genomic location of each non-core region are not reflected in the plot. The RT and MLST_8_ data^10^,^12^ for each genome are listed after the strain ID. ND - not determined.

To infer whether potential functional differences exist between type III and other *P. acnes* lineages, four publicly available type III genomes (HL201PA1, JCM18909, PMH5 and PMH7), including two from PMH lesional skin (PMH5, PMH7), were compared with *P. acnes* genomes from type I (n = 69) and type II (n = 12) lineages, respectively (Fig. 3). Comparative genomic analyses revealed the presence of eight non-core regions found exclusively in all four type III genomes (type III-specific regions; Fig. 3; Supplementary Table S2), and a further two regions (regions 19 and 22) specific to three of the four type III genomes (HL201PA1, PMH5 and PMH7) (Fig. 3; Supplementary Table S2). Additionally, we identified regions found in type III strains that were also shared by a small number of other *P. acnes* strains, including those from the type IB and IC phylogroups (Supplementary Table S2). Genomic regions primarily found in type III strains (with some minor exceptions) accounted for over 109 kb, and contained 111 predicted open reading frames (ORFs) with putative functions in metabolism, transport and transcriptional regulation, as well as phage proteins and a large number of predicted proteins of unknown function (Supplementary Table S2). Within region 18, which is exclusive to all four type III genomes, two putative type II secretion system (T2SS) components and a putative operon of tight adherence (Tad) genes, consisting of pilus components TadE and CpaB, as well as prepilin peptidase A24, were identified (Supplementary Table S2). However, this region shows no homology with a previously described Tad locus that occurs in a plasmid from the type IA_2_ strain HL096PA1^13^.

Compared to type I and type II strains, type III strains were found to lack a number of genomic regions, with a cumulative length of 69.6 kb (type III-absent regions) (Supplementary Table S3). In particular, all four of the type III strains lack 17 genomic regions present in other *P. acnes* type I and type II strains (Fig. 3). Additionally, three of the four type III genomes (HL201PA1, PMH5 and PM7) are missing another five genomic regions that are present in all other *P. acnes* phylogroups, as well as type III strain JCM18909 (Fig. 3; Supplementary Table S3). A further four regions absent in all type III genomes were also missing from a small number of other *P. acnes* strains, including those from the type IB and IC phylogroups similar to the findings of Tomida *et al*.^16^ (Fig. 3; Supplementary Table S3). ORFs encoding dimethyl sulfoxide reductase, hyaluronate lyase, class IIb bacteriocins, cobalamin biosynthesis, iron and other ABC transporter systems and maltose metabolism were found to be absent in type III strains. Furthermore, all type III strains appear to have an inactive CRISPR/cas system that we previously described in this bacterium^12^,^16^.

## Discussion

The categorical demonstration in this study that type III strains are strongly associated with PMH raises the possibility that this lineage may influence the development of the condition, although this yet remains to be established. Our determination that the AFLP DNA group 3 strains previously isolated from PMH patients in the Netherlands also appear to belong to the type III lineage, and not a novel *Propionibacterium* species as suggested^3^, adds additional weight to support this view, as does the recent isolation of type III strains from the lower back of two patients with PMH in Aalborg, Denmark^15^. Furthermore, the observation that type III strains recovered from PMH lesions could not be identified as *P. acnes* using the current Rapid ID 32A multi-test identification system, unlike strains of types I and II, is also consistent with the results previously described for AFLP DNA group 3 strains isolated from PMH patients^3^. While the high rate of *P. acnes* recovery, especially type III, from PMH lesions does not in itself prove causality, the circumstantial evidence linking this bacterium to the aetiology of the condition is strong, especially the low rate of recovery of *P. acnes* form peri-lesional skin and the resolution of symptoms (repigmentation) with anti-bacterial therapeutics^5^,^6^,^7^,^8^. Other factors are, however, also likely to be important in the development of PMH including genetic factors, hormonal influences and the host response to the skin microbiota, which may prevent disease in some individuals but not in others.

The association of type III strains with PMH provides evidence that this genetic division may be linked to human disease, and not represent ‘true’ commensals solely associated with skin health as tentatively suggested previously^10^,^11^. The marked presence of type III strains in PMH lesions is remarkable and in stark contrast to the *P. acnes* types isolated from the skin condition acne vulgaris, which appears to be predominately associated with epidemic lineages from the type IA_1_ grouping^9^,^10^,^11^,^12^,^17^. This may help to explain why no clear correlation exists between acne and the development of PMH^3^. Type III strains are present on normal skin and have been isolated from the face and other sites^10^, but previous 16S rDNA-based metagenomic analysis of facial skin from healthy individuals and acne patients in the United States revealed that type III strains are in low abundance in this region^12^. The overwhelming majority of strains identified in this previous acne study belonged to the type I division with <1% of all clones containing the characteristic type III 16S rDNA mutation^12^. Against this background, the rate of detection of type III strains from PMH patients, particularly using a culture-based approach, is striking. This association between type III strains and PMH is further supported by the isolation of type III strains from other PMH cohorts in Europe^3^,^15^.

The previous observation of a relatively higher rate of recovery of type III strains from intervertebral disc material (excised disc protrusion) removed during spinal surgery is now also interesting in light of our results^18^. These earlier studies led to the proposal that chronic, low-grade *P. acnes* infection may play a role in the development of severe sciatica^18^,^19^,^20^,^21^. However, this is a controversial idea, and some studies suggest that the presence of the bacterium in such material simply reflects contamination of the surgical wound site from the surrounding skin on the lower back, even after pre-operative skin antisepsis and antimicrobial prophylaxis^22^,^23^,^24^,^25^. While the abundance of type III strains within and between different body sites of individuals currently remains poorly defined, the isolation of type III strains from such contaminated material would tentatively suggest that the relative proportion of type III colonizing the skin on the lower back, and possibly other regions of the trunk, may be higher with this lineage than at other sites which may explain, at least in part, why the trunk is affected by PMH and not the face.

To better understand the nature of the strains associated with PMH we performed high resolution MLST on 24 isolates. Although the type III phylogroup is globally disseminated within the human population^10^,^11^, our analysis revealed that the type III STs recovered from lesional and paired non-lesional skin of the PMH patients are distinct from those so far recovered from PMH lesions, facial skin, and other clinical samples in Europe. Some of these STs (particularly ST75 and ST77) have, however, been previously described in relation to non-acneic and normal skin sampled from subjects in this region of Brazil^10^,^11^. In contrast, the PMH isolates recovered from patients in Aalborg, Denmark, had the genotype ST33, which has been identified in association with spinal disc material removed from patients undergoing spinal surgery in Birmingham, UK^10^,^11^,^14^, as well as from the normal facial skin of a subject in Hungary^10^; this provides tentative evidence for the widespread dissemination of this particular type III ST in Europe and possibly further afield. Interestingly, ST77, while currently only described in association with Brazilian skin, is the founder genotype for ST33 and ST81 currently identified in Europe^10^. Normally, founding genotypes are more prevalent within the population and widely disseminated. Further epidemiological studies will, therefore, enable us to investigate how geographically widespread the different type III STs identified in this study actually are, and whether they are enriched in the skin microbiota of Brazilian individuals versus those in Europe and elsewhere, particularly those with PMH, which would initially appear to be the case. In contrast to the type III strains identified in this study, MLST analysis of other lesional isolates revealed STs that are common and globally disseminated on the skin of the human population^10^,^11^. While the overall recovery of such strains from PMH lesions was low compared with type III, and not statistically significant, their overall rate of isolation was still higher compared to non-lesional skin; the genome counts of *P. acnes* in lesional skin from such samples were sometimes very high in comparison to the adjacent non-lesional skin. The exact role such phylogroups may play in influencing the development of PMH in these particular cases therefore remains unclear. In acneic skin, we also see a similar pattern where phylogroups other than type IA_1_ are isolated, but at significantly reduced rates.

The strong association of *P. acnes* type III strains with PMH may reflect distinct genomic and transcriptomic properties of strains from this genetic division which are important in the interaction with the human host. Previous phenotypic studies with a limited number of type III strains have found that they can adopt a filamentous morphology, and do not produce cell surface dermatan sulphate-binding adhesins, sialidase or β-haemolytic activity, unlike the type IA lineage, but our understanding of their functional potential is still very limited^9^,^11^,^14^,^17^. Although the primary focus of this study was epidemiological, we also conducted a comparative genomic analysis to investigate whether differences between type III and type I and II strains could provide tentative clues as to why this lineage in particular accumulates within PMH lesional skin, but is not associated with acne. Within type III strains, an inactive CRISPR/cas system is present which may be relevant to the acquisition of genetic loci that contribute to virulence, adaptation and disease association^12^. In light of this, the observation of the type III-specific region 18 containing T2SS genes, more commonly found in Gram-negative bacteria, was interesting. Such genes have been linked to a broad range of infections and disease and could provide a mechanism whereby secreted proteins promote pathogen colonisation and contribute to host tissue damage^26^. These T2SS genes are in close genomic proximity to a putative Tad locus, a virulence factor in several bacterial pathogens, and together may form a potential type III-specific pathogenicity island. This region, however, shows no homology with a previously described Tad locus found in acne-associated strains from the type IA_1_ clade^13^. Furthermore, two genomic islands, known as loci 1 and 2, that contain genes proposed to enhance virulence via increased bacterial adhesion and host immune response, and also present in acne-associated strains, are absent from the type III strains examined. Other notable differences include the presence of a large number of type III-specific hypothetical proteins of unknown function, and the presence and absence of specific ABC transporter proteins which are important in bacterial cell viability and physiology, as well as pathogenicity. A dimethyl sulfoxide reductase gene, which may influence growth rate under anaerobic conditions, was also missing from type III strains, as was a previously described hyaluronate lyase^27^. We also observed other differences in relation to genes involved in various metabolic processes, including sugar utilization. As the skin is an ecosystem of diverse habitats, these differences may all be important in relation to specific niche adaptation and colonization of different skin sites. Combined with functional differences, this could potentially explain their association with PMH on the truck, but not facial acne. Our genomic comparisons also revealed that the Japanese facial isolate JCM18909^28^ lacked a number of genomic regions found in the other three type III genomes, but shared similarities with *P. acnes* isolates belonging to the type I and type II lineages which are abundant on facial skin^12^. This could indicate that isolates belonging to this lineage are equipped with unique metabolic properties to aid adaptation to the niche from where they were isolated, but this suggestion is currently tentative due to the assembly of JCM18909 which has a much lower N50 than the other genomes. Genome sequencing of more type III and PMH-derived isolates, as well as further studies incorporating transcriptional and functional characterisation of type III-specific genomic elements, will undoubtedly provide additional insight into the role played by type III strains in PMH.

In conclusion, while the underlying aetiology of PMH is still not firmly established, in this study we have conclusively demonstrated a high rate of *P. acnes* type III in PMH lesional skin using a culture-based method for the detection of viable bacteria. In contrast, there was little evidence of *P. acnes* in adjacent non-lesional skin based on plate culture of punch biopsies, which reflected lower levels of the bacterium in these samples as measured by qPCR analysis. This is consistent with the study of McGinley *et al*.^29^ who found that the prevalence of *P. acnes* on the lower back of the trunk was significantly reduced when compared with the upper trunk and face where levels of sebum production are higher. This appears to suggest enrichment or abnormal overgrowth of the bacterium at lesional skin sites on the trunk of patients, although the underlying factor(s) responsible for this is currently unclear. Future non-culture-based metagenomic studies will better capture the overall multi-phyletic community level structure and functional potential of the skin microbiota in PMH patients, especially alongside healthy non-PMH control subjects, and may provide deeper insights into the prevalence and role of type III and other phylogroups in the condition.

## Materials and Methods

### Patients and sampling

The *P. acnes* isolates analysed in this study were previously recovered from 34 PMH patients attending a dermatology outpatient clinic at the Oswalso Cruz University Hospital, Recife, Brazil from March-to-May 2008, as originally described by Cavalcanti *et al*.^4^. The original study was approved by the Ethics Commission at Oswalso Cruz University Hospital (no. 147/2007) and written informed consent was obtained from all subjects. Experiments were performed in accordance with relevant approved guidelines and regulations. In brief, PMH was characterized by the presence of asymptomatic hypochromic macules on the back with a tendency to spread to the middle of the back. The patients were aged between 18 and 42 years with the majority being female (75%). Most patients (55%) had no family history of PMH and phototypes IV and V were the most common reflecting this region of Brazil. The study exclusion criteria included patients with acne on the back, the presence of dermatosis on the truck, lactation, or the use of antimicrobials during the three months prior to consultation. All patients were submitted to a dermatological naked eye exam by three dermatologists and a Wood’s lamp exam in a darkroom to detect the presence of follicular fluorescence due to bacterial porphyrin production. Lesional and non-lesional skin from the back was identified using the Wood’s lamp and naked eye examination, and fragments from the same anatomical site removed with a minimum distance of 1 cm using a 4 mm skin punch.

### Quantitative Real Time PCR (qPCR)

*P. acnes* genome copy number in DNA extracted from the macerates of lesional and non-lesional skin was determined in triplicate by qPCR as described previously^4^.

### Bacterial strains and culture

The original lesional and non-lesional skin fragments were homogenized and the macerates cultured under anaerobic conditions as previously described^4^. Multiple colonies of presumptive *P. acnes* were initially identified based on a combination of Gram staining and biochemical analysis (production of indole, nitrate and catalase and esculin degradation). All bacterial strains were maintained at −80 °C in brain heart infusion (BHI) broth containing 12% (v/v) glycerol. Prior to molecular analysis, anaerobic isolates were cultured on anaerobic horse blood agar plates (Oxoid Ltd., Hampshire, United Kingdom) at 37 °C in an anaerobic cabinet (Mark III; Don Whitley Scientific) under an atmosphere of 10% H_2_, 10% CO_2_, and 80% N_2_ for a minimum of five days. For stringency in our results, samples were only classified as culture positive if they also had a corresponding *P. acnes* genome copy number that was above the third quartile (Q3) + 3 x interquartile range (IQR) value obtained with non-lesional samples that were culture-negative.

### Multiplex PCR analysis of isolates

Bacterial genomic DNA was purified using a QIAamp DNA mini kit (QIAGEN, United Kingdom) and PCR amplification was carried out using a MultiGene thermocycler (Labnet International). Initial typing of multiple *P. acnes* isolates was performed using a rapid multiplex PCR assay as previously described^30^. This assay confirms *P. acnes* species identity and phylogeny of isolates (types IA_1_, IA_2_, IB and IC, II and III) based on primer sets targeting the 16S rRNA gene as well as a series of protein-encoding genes.

### MLST

High resolution typing of isolates was carried out using an eight gene scheme (4253 bp) previously described for this bacterium (MLST_8_)^10^. Sequencing was performed on an ABI PRISM genetic analyser (Life Technologies). Allele and sequence types (ST) were compared to those within the *P. acnes* MLST database at http://pubmlst.org/pacnes/.

### Bioinformatic analyses

Core and non-core regions of 85 *P. acnes* genomes were calculated as previously described^16^. Briefly, core regions were calculated by mapping 84 genome sequences against the reference genome KPA171202 using Nucmer^31^. Regions of KPA171202 that aligned with all 84 genomes were identified within the 84 “.coords” output files, and the corresponding aligned regions in all 85 genomes were extracted. For each set of core regions of a genome, 124,731 single nucleotide polymorphisms (SNPs) relative to KPA171202 were identified and concatenated into a single sequence. MEGA v5 was then used to calculate a neighbour-joining phylogenetic tree based on the p-distances between each of the 85 concatenated SNP sequences^32^. The bootstrap tree inferred from 200 replicates was used. To determine non-core regions across the 85 isolates, Nucmer was used to align the KPA171202 genome to one of the 84 other genomes in order to determine the regions unique to the latter isolate. These regions were then added to the KPA171202 genome to form a second reference sequence that was aligned to another of the remaining 83 genomes. Iterating the above process for all the remaining isolates resulted in a pan-genome of the 85 isolates. The core regions were subtracted from the pan-genome to obtain a set of noncore regions. Protein encoding sequences in non-core regions were predicted by GeneMark.hmm^33^ using KPA171202 as a reference. Annotations were performed using BlastX against the NCBI RefSeq protein database.

### Biochemical analysis

Biochemical analysis was carried out using a RAPID ID 32A biochemical profiling kit according to the manufacturer’s instructions (bioMérieux, UK).

### Statistical analysis

Comparison of phylogroups between paired samples was performed using McNemar’s test for paired nominal data (two-tailed). Genome copy number between paired samples was compared using the non-parametric Wilcoxon Signed Rank test (two-tailed).

## Additional Information

**How to cite this article**: Barnard, E. *et al*. Strains of the *Propionibacterium acnes* type III lineage are associated with the skin condition progressive macular hypomelanosis. *Sci. Rep.* **6**, 31968; doi: 10.1038/srep31968 (2016).

## Supplementary Material

Supplementary Information

## Figures and Tables

**Table 1:** Culture and multiplex PCR results for paired lesional and non-lesional skin from patients with PMH.

Patient ID #	Culture result		Multiplex PCR results from multi-isolate analysis	
	Lesion	Non-lesion	Lesion	Non-lesion
BR-1	+	−	IA_2_	−
BR-2	−	−	−	−
BR-3	+	−	IA_1_	−
BR-4	+	−	IA_1_	−
BR-5	+	−	IA_2_	−
BR-6	−	−	−	−
BR-7	+	−	II	−
BR-8	−	−	−	−
BR-9	+	−	III	−
BR-10	+	−	II	−
BR-11	−	−	−	−
BR-12	+	−	IB	−
BR-13	+	−	III	−
BR-14	+	−	III	−
BR-15	−	−	−	−
BR-16	+	−	III	−
BR-17	+	−	III	−
BR-18	+	−	III	−
BR-19	−	−	−	−
BR-20	−	+	−	III/IA_1_
BR-21	+	−	III	−
BR-22	+	−	III	−
BR-23	+	−	IA_1_	−
BR-24	+	−	II	−
BR-25	+	−	IB/II	−
BR-26	+	−	II	−
BR-27	+	−	III	−
BR-28	+	−	IA_1_	−
BR-29	+	−	III	−
BR-30	+	−	III/IA_2_	−
BR-31	+	−	IA_2_	−
BR-32	+	−	III	−
BR-33	+	−	III	−
BR-34	+	−	III	−
