# Supplementary material for: Strains of the Propionibacterium acnes type III lineage are associated with the skin condition progressive macular hypomelanosis
Source: Sci Rep. 2016 Aug 24;6:31968. doi: 10.1038/srep31968 (PMC4995408; doi:10.1038/srep31968)
Supplement: Supplementary Information [file srep31968-s1.pdf]

Supplementary information for:

Title: Strains of the *Propionibacterium acnes* type III lineage are associated with the skin condition Progressive Macular Hypomelanosis

Authors: Emma Barnard, Jared Liu, Eliza Yankova, Silvana M. Cavalcanti, Marcelo Magalhães, Huiying Li, Sheila Patrick and Andrew McDowell

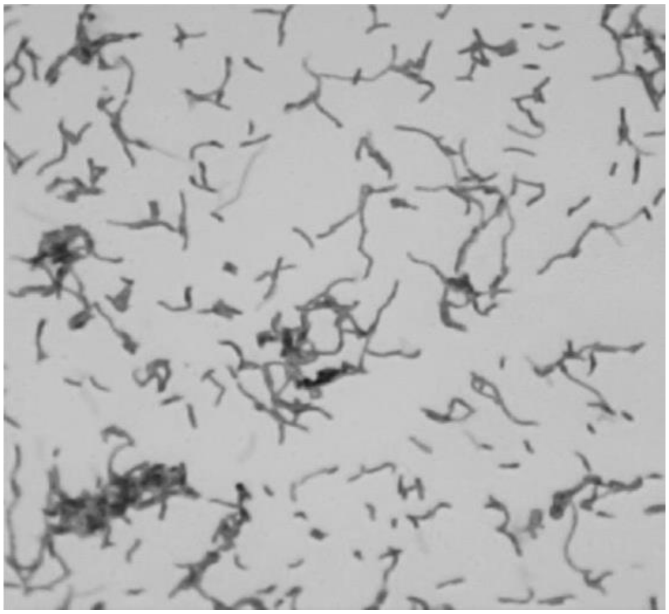

Figure S1. Gram stain of a type III isolate from PMH lesional skin.
Micrograph (obj. x100)

Strain ID	16S Sequence 5'-3'	*P. acnes* type
JCM18909	…GTGGCGAGCCTGTAAGGGTGAGCGAAT…	Type III
Ribotype1	…GTGGCGAGCCTGTGAGGGTGAGCGAAT…	Type I
Ribotype2	…GTGGCGAGCCTGTGAGGGTGAGCGAAT…	Type II
Ribotype3	…GTGGCGAGCCTGTGAGGGTGAGCGAAT…	Type I
Ribotype4	…GTGGCGAGCCTGTGAGGGTGAGCGAAT…	Type I
Ribotype5	…GTGGCGAGCCTGTGAGGGTGAGCGAAT…	Type I
Ribotype6	…GTGGCGAGCCTGTGAGGGTGAGCGAAT…	Type II
Ribotype7	…GTGGCGAGCCTGTGAGGGTGAGCGAAT…	Unknown
Ribotype8	…GTGGCGAGCCTGTGAGGGTGAGCGAAT…	Type I
Ribotype9	…GTGGCGAGCCTGTAAGGGTGAGCGAAT…	Unknown
Ribotype10	…GTGGCGAGCCTGTGAGGGTGAGCGAAT…	Unknown

1256 1268 1280

Figure S2. RT9 containing the type III mutation G1268A is found with low abundance on facial skin. Alignment of the 16S rDNA sequence from type III genome JCM18909 with the ten most common 16S ribotypes (top-ten) found on healthy and acneic facial skin, shows that RT9 contains the type III-specific mutation G1268A. 16S rDNA clone analysis revealed that RT9 is found in low abundance on facial skin, with <1% of top-ten clones (Fitz-Gibbon *et al.*, 2013).

Table S1. Rapid ID 32A multi-test identification results for PMH isolates

Isolate (type)	Significant taxa	% identity	Profile
ATCC6919 (IA_1)	*Propionibacterium acnes*	99.9	2000 3206 04
hdn-1 (IA_1)	*Propionibacterium acnes*	99.9	0003 3206 04
NCTC10390 (II)	*Propionibacterium acnes*	99.9	2403 3336 04
BR-26 (II)	*Propionibacterium acnes*	99.9	2403 3336 04
BR-16 (III)	*Clostridium tetani*	97.5	2000 2000 00
BR-29 (III)	*Eggerthella lenta*<br>*Propionibacterium acnes*<br>*Clostridium sordellii*<br>*Veillonella spp*<br>*Clostridium bifermentans*	65.3<br>9.8<br>7.4<br>6.0<br>5.3	2000 3200 00
BR-34 (III)	*Fusobacterium nucleatum*<br>*Clostridium tyrobutyricum*<br>*Clostridium tetani*<br>*Clostridium bifermentans*<br>*Veillonella spp*	42.1<br>23.6<br>14.0<br>8.8<br>8.3	0000 3000 00

Table S2. Type III-specific genes. Genes in 23 type III non-core regions that are specific to type III strains or shared with only a small number of other *P. acnes* strains are listed. Two regions (19 and 22) were found to be specific to three of the four type III genomes.

Description	Length (AA)	e-value	Non-core region	Length (nt)	Also found in:
DNA processing protein DprA [Propionibacterium]	179	1.14E-108	1	541	KPA171202<br>6609<br>HL030PA1<br>SK187
transposase [Propionibacterium]	97	9.93E-62	2	13,328	KPA171202<br>6609<br>HL030PA1<br>SK187
transposase [Propionibacterium]	174	5.51E-124			
lanthionine biosynthesis protein [Propionibacterium]	629	0			
lanthionine biosynthesis protein [Propionibacterium]	314	0			
hypothetical protein [Propionibacterium]	277	0			
hypothetical protein [Propionibacterium acnes]	255	6.4E-179			
hypothetical protein [Propionibacterium]	196	5.9E-141			
cyclodehydratase [Propionibacterium]	531	0			
hypothetical protein [Propionibacterium]	152	1.61E-102			
hypothetical protein	48	$> 10^{-5}$			
hypothetical protein [Propionibacterium]	71	1.06E-43			
transposase [Propionibacterium]	97	9.93E-62			
hypothetical protein [Propionibacterium acnes]	63	1.47E-34	3	2,272	KPA171202<br>6609<br>HL030PA1<br>SK187
hypothetical protein [Propionibacterium acnes]	103	1.66E-53			
hypothetical protein [Propionibacterium]	89	2.49E-56			
hypothetical protein [Propionibacterium]	226	6.08E-127			
PREDICTED: ankyrin repeat domain-containing protein 24 [Gorilla gorilla gorilla]	71	$> 10^{-5}$			
hypothetical protein	17	$> 10^{-5}$	4	1,870	KPA171202<br>6609<br>HL030PA1<br>HL097PA1<br>PRP-38
hypothetical protein	207	$> 10^{-5}$			
LacI family transcriptional regulator [Propionibacterium]	339	0	5	32,894	KPA171202<br>6609<br>HL030PA1
beta-glucanase [Propionibacterium]	445	0			
alpha-galactosidase [Propionibacterium acnes]	705	0			
sugar-binding protein [Propionibacterium]	436	0			
ABC transporter permease [Propionibacterium]	300	0			
sugar ABC transporter permease [Propionibacterium acnes]	271	0			
microcystinase C [Propionibacterium]	487	0			
ROK family transcriptional regulator [Propionibacterium]	397	0			
peptide ABC transporter substrate-binding protein [Propionibacterium]	598	0			
diguanylate cyclase [Propionibacterium]	305	0			
peptide ABC transporter permease [Propionibacterium]	313	0			
peptide ABC transporter ATP-binding protein [Propionibacterium]	684	0			

transcriptional regulator [Propionibacterium]	312	0			
alpha-L-fucosidase [Propionibacterium]	436	0			
inosine-uridine preferring nucleoside hydrolase [Propionibacterium]	231	1.59E-158			
TetR family transcriptional regulator [Propionibacterium]	219	9.64E-150			
ABC transporter [Propionibacterium acnes]	221	2.51E-149			
ABC transporter [Propionibacterium acnes]	65	4.44E-17			
ABC transporter [Propionibacterium acnes]	284	4.3E-179			
iron ABC transporter ATP-binding protein [Propionibacterium]	621	0			
permease [Propionibacterium]	197	1.92E-133			
ABC transporter ATP-binding protein [Propionibacterium]	510	0			
cobalt ABC transporter permease [Propionibacterium]	272	0			
membrane protein [Propionibacterium]	138	1.48E-87			
hypothetical protein [Propionibacterium]	73	3.32E-42			
hypothetical protein [Propionibacterium]	227	7.56E-158			
membrane protein [Propionibacterium]	234	1.01E-159			
ABC transporter permease [Propionibacterium]	455	0			
ABC transporter ATP-binding protein [Propionibacterium]	244	4.57E-172			
diaminohydroxyphosphoribosylaminopyrimidine deaminase [Propionibacterium]	354	0			
dihydrodipicolinate synthase family protein [Propionibacterium]	195	1.68E-139			
hypothetical protein [Propionibacterium]	191	5.18E-133			
hypothetical protein [Propionibacterium]	70	4.6E-40			
hypothetical protein [Propionibacterium]	250	0			
VWA domain-containing protein [Propionibacterium]	316	0			
VWA domain-containing protein [Propionibacterium]	323	0			
hypothetical protein [Propionibacterium acnes]	162	7.26E-109	6	11,740	HL025PA1
hypothetical protein [Propionibacterium]	295	0			
ATPase AAA [Propionibacterium acnes]	320	0			
membrane protein [Propionibacterium]	296	0			
hypothetical protein [Propionibacterium]	482	6.11E-161			
membrane protein [Propionibacterium]	342	0			
hypothetical protein [Propionibacterium]	104	1.15E-65			
hypothetical protein [Propionibacterium]	172	6.76E-118	7	831	HL025PA1
hypothetical protein [Cellulosimicrobium cellulans]	61	$> 10^{-5}$			
hypothetical protein_partial [Propionibacterium sp. 409-HC1]	27	1.81E-07			
hypothetical protein [Propionibacterium]	72	6.68E-38	8	816	HL025PA1
hypothetical protein	69	$> 10^{-5}$			
hypothetical protein	31	$> 10^{-5}$	9	581	HL045PA1<br>HL027PA1
ABC transporter permease [Propionibacterium]	125	4.91E-81			
ABC transporter substrate-binding protein [Propionibacterium humerusii]	115	2.54E-67	10	753	HL097PA1<br>PRP-38<br>HL050PA1
ABC transporter substrate-binding protein [Propionibacterium acnes]	92	2.23E-28	11	2,300	HL097PA1<br>PRP-38
hypothetical protein [Propionibacterium acnes]	45	3.26E-16			

hypothetical protein	468	> 10^{-5}			
restriction endonuclease subunit S [Propionibacterium]<br>DEAD/DEAH box helicase [Propionibacterium acnes]<br>DEAD/DEAH box helicase [Propionibacterium acnes]<br>hypothetical protein	27<br>107<br>924<br>45	5.81E-10<br>1.86E-63<br>0<br>> 10^{-5}	12	3,599	HL097PA1<br>PRP-38
hypothetical protein<br>hypothetical protein [Propionibacterium acnes]<br>RHS repeat-associated core domain-containing protein [Propionibacterium acnes]<br>pyrophosphorylase [Propionibacterium acnes]<br>hypothetical protein [Propionibacterium acnes]<br>hypothetical protein [Propionibacterium acnes]<br>hypothetical protein [Propionibacterium acnes]<br>FHA domain-containing protein [Propionibacterium acnes]<br>cell division protein FtsK [Propionibacterium acnes]<br>hypothetical protein [Pseudomonas mediterranea]<br>hypothetical protein PRUPE_ppa025858mg_partial [Prunus persica]	15<br>62<br>469<br>93<br>180<br>721<br>433<br>673<br>624<br>169<br>82	-<br>5.96E-36<br>0<br>8.76E-59<br>1.56E-121<br>0<br>0<br>0<br>0<br>> 10^{-5}<br>> 10^{-5}	13	12,625	-
hypothetical protein [Propionibacterium]<br>hypothetical protein [Propionibacterium acnes]<br>hypothetical protein [Propionibacterium acnes]<br>hypothetical protein [Pseudophaeobacter arcticus]<br>hypothetical protein [Propionibacterium acnes]<br>hypothetical protein [Propionibacterium acnes]<br>ABC transporter substrate-binding protein [Propionibacterium acnes]	58<br>156<br>134<br>307<br>94<br>173<br>451	5.37E-26<br>3.04E-111<br>1.29E-84<br>3.71E-74<br>4.26E-61<br>5.35E-126<br>0	14	7,005	-
DEAD/DEAH box helicase [Propionibacterium acnes]	477	0	15	1,433	HL097PA1<br>PRP-38
hypothetical protein<br>hypothetical protein [Herpetosiphon geysericola]<br>ABC transporter ATP-binding protein [Propionibacterium acnes]<br>conjugal transfer protein TraL [Propionibacterium acnes]<br>hypothetical protein [Propionibacterium acnes]<br>beta-glucosidase [Propionibacterium acidipropionici]<br>hypothetical protein [Saccharothrix syringae]<br>hypothetical protein<br>hypothetical protein [Propionibacterium acnes]<br>hypothetical protein [Propionibacterium acnes]<br>hypothetical protein<br>sugar ABC transporter permease [Propionibacterium acnes]<br>sugar ABC transporter permease [Propionibacterium acnes]<br>ABC transporter substrate-binding protein [Propionibacterium acnes]<br>hypothetical protein [Propionibacterium acnes]<br>hypothetical protein [Propionibacterium humerusii]<br>short-chain dehydrogenase [Propionibacterium acnes]<br>DNA polymerase [Arcanobacterium haemolyticum]<br>DNA polymerase [Mobiluncus curtisii]	125<br>128<br>106<br>408<br>136<br>600<br>39<br>50<br>455<br>595<br>40<br>275<br>280<br>425<br>348<br>69<br>74<br>364<br>55	> 10^{-5}<br>> 10^{-5}<br>7.43E-55<br>0<br>2.75E-76<br>0<br>> 10^{-5}<br>> 10^{-5}<br>0<br>0<br>> 10^{-5}<br>0<br>0<br>0<br>0<br>1.07E-24<br>1.05E-35<br>0<br>6.8E-16	16	49,352	-

hypothetical protein [Actinomyces turicensis]	44	9.37E-10			
hypothetical protein [Propionibacterium acnes]	181	1.32E-129			
nuclease [Dermabacter hominis]	228	1.57E-139			
hypothetical protein [Propionimicrobium lymphophilum]	150	1.05E-81			
hypothetical protein [Propionibacterium acnes]	134	2.66E-90			
hypothetical protein [Corynebacterium vitaeruminis]	54	4.09E-10			
hypothetical protein [Propionibacterium acnes]	114	1.03E-77			
hypothetical protein [Mobiluncus curtisii]	243	4.74E-109			
hypothetical protein [Propionibacterium avidum]	106	1.22E-50			
hypothetical protein [Propionibacterium acnes]	75	2.13E-42			
chromosome segregation ATPase [Propionibacterium avidum]	393	0			
chromosome segregation ATPase [Propionibacterium avidum]	149	4.79E-72			
transcriptional regulator [Propionibacterium acnes]	69	5.05E-41			
hypothetical protein [Propionibacterium acnes]	102	3.99E-69			
mechanosensitive ion channel protein MscS [Photobacterium]	51	$> 10^{-5}$			
hypothetical protein [Propionibacterium acnes]	143	3.1E-98			
hypothetical protein [Propionibacterium acnes]	129	2.14E-86			
hypothetical protein [Propionibacterium acnes]	163	7.35E-117			
hypothetical protein [Propionibacterium acnes]	91	3.63E-59			
hypothetical protein [Propionibacterium acnes]	118	2.43E-77			
hypothetical protein	57	$> 10^{-5}$			
hypothetical protein [Trueperella pyogenes]	52	8.41E-09			
transposase [Corynebacterium tuscaniense]	61	4.52E-18			
transposase [Corynebacterium]	225	6.83E-132			
transposase [Actinomyces meyeri]	118	1.12E-58			
hypothetical protein [Arcanobacterium haemolyticum]	191	1.09E-81			
hypothetical protein [Propionibacterium sp. oral taxon 192]	35	1.91E-11			
hypothetical protein [Propionibacterium sp. oral taxon 192]	50	5.31E-12			
hypothetical protein [Propionibacterium acnes]	59	9.43E-36			
hypothetical protein [Propionibacterium acnes]	543	0			
hypothetical protein [Propionibacterium acnes]	352	0			
hypothetical protein [Propionibacterium acnes]	380	0			
transposase [Propionibacterium acnes]	101	3.04E-56			
transposase [actinobacterium LLX17]	319	1.68E-109			
hypothetical protein [Propionibacterium acnes]	100	5.26E-64			
hypothetical protein [Propionibacterium acnes]	152	9.4E-94			
transporter [Brevibacterium linens]	93	1.14E-22			
transporter [Brevibacterium linens]	313	1.28E-95			
hypothetical protein [Propionibacterium acnes]	194	8.56E-139			
4-aminobutyrate aminotransferase [Propionibacterium acnes]	419	0			
inorganic polyphosphate kinase [Streptomyces niveus]	270	2.6E-81			
hypothetical protein [Propionibacterium acnes]	341	0			
hypothetical protein [Propionibacterium acnes]	532	0			
hypothetical protein	40	$> 10^{-5}$			
dihydrolipoyl dehydrogenase [Actinomyces europaeus]	65	$> 10^{-5}$	17	7,592	-

ABC transporter substrate-binding protein [Propionibacterium acnes]	544	0			
ABC transporter permease [Propionibacterium acnes]	321	0			
hypothetical protein [Propionibacterium acnes]	335	0			
ABC transporter [Propionibacterium acnes]	338	0			
ABC transporter [Propionibacterium acnes]	259	0			
hypothetical protein_partial [Propionibacterium acnes]	159	9.96E-105			
hypothetical protein	53	> 10^{-5}			
peptidase A24 [Propionibacterium humerusii]	55	4.63E-12	18	17,722	-
hypothetical protein [Propionibacterium acnes]	193	6.28E-133			
hypothetical protein [Propionibacterium acnes]	256	0			
pilus assembly protein CpaB [Propionibacterium acnes]	242	5.7E-168			
transcriptional regulator [Propionibacterium humerusii]	497	0			
pilus assembly protein TadE [Propionibacterium acnes]	140	3.57E-79			
secretion protein [Propionibacterium acnes]	437	0			
type II secretion protein F [Propionibacterium acnes]	307	0			
type II secretion protein F [Propionibacterium acnes]	301	0			
two-component sensor histidine kinase [Propionibacterium acnes]	390	0			
DNA-binding response regulator [Propionibacterium acnes]	231	6.33E-164			
hypothetical protein [Propionibacterium humerusii]	74	1.85E-26			
hypothetical protein [Propionibacterium acnes]	361	4.61E-95			
hypothetical protein [Propionibacterium]	81	1.34E-13			
hypothetical protein_partial [Propionibacterium acnes]	144	5.99E-93			
MFS transporter [Propionibacterium acnes]	526	0			
hypothetical protein [Propionibacterium acnes]	335	0			
inositol 2-dehydrogenase [Propionibacterium acnes]	256	0			
AP endonuclease [Actinomyces johnsonii]	383	0			
hypothetical protein [Propionibacterium acnes]	86	6.84E-54			
DeoR family transcriptional regulator [Propionibacterium propionicum]	53	0.0000027			
hypothetical protein	54	> 10^{-5}			
hypothetical protein [Propionibacterium acnes]	172	5.92E-121	19	2,027	-
hypothetical protein [Propionibacterium acnes]	82	2.4E-51			
hypothetical protein [Propionibacterium acnes]	104	7.36E-70			
hypothetical protein	42	> 10^{-5}			
lactate dehydrogenase [Nocardioides halotolerans]	62	8.35E-09	20	3,137	-
hypothetical protein [Propionibacterium acnes]	300	0			
hypothetical protein	387	> 10^{-5}			
HNH nuclease [Propionibacterium acnes]	347	0	21	8,797	-
hypothetical protein [Propionibacterium acnes]	231	2.43E-142			
hypothetical protein [Propionibacterium acnes]	116	1.58E-74			
hypothetical protein [Propionibacterium acnes]	258	4.62E-152			
hypothetical protein [Propionibacterium granulosum]	409	8.69E-103			
hypothetical protein [Propionibacterium acnes]	145	6.23E-79			
restriction endonuclease subunit M [Salinispora pacifica]	39	7.63E-13	22	680	-
hypothetical protein [Propionibacterium acnes]	43	2.13E-19	23	734	-
hypothetical protein_partial [Propionibacterium acnes]	69	> 10^{-5}			

Table S3. Type III-absent genes. Genes from 26 type III-absent regions. Some regions are also absent in other *P. acnes* strains. Five regions (5, 7, 9, 11, and 21) were absent in only three of the four type III genomes.

Description	Length (AA)	e-value	Non-core region	Length (nt)	Also absent in:
ABC transporter substrate-binding protein [Propionibacterium acnes]	414	0	1	2,691	-
ABC transporter substrate-binding protein [Propionibacterium]	402	0			
LacI family transcriptional regulator [Propionibacterium]	317	0	2	4,336	HL025PA1 HL097PA1 PRP-38
dehydrogenase [Propionibacterium acnes]	144	9.52E-71			
dehydrogenase [Propionibacterium acnes]	220	2.11E-154			
sugar phosphate isomerase [Propionibacterium]	332	0			
protein iolH [Propionibacterium]	291	0			
dimethyl sulfoxide reductase subunit C [Propionibacterium acnes]	332	0	3	3,559	-
dimethyl sulfoxide reductase subunit B [Propionibacterium acnes]	213	1.25E-156			
dimethyl sulfoxide reductase subunit A [Propionibacterium acnes]	584	0			
phosphoesterase [Propionibacterium acnes]	179	1.79E-117	4	538	-
hypothetical protein [Propionibacterium]	61	1.10E-35	5	878	-
hypothetical protein [Propionibacterium humerusii]	57	2.95E-27			
htaa_partial [Propionibacterium acnes]	295	0	6	4,402	-
magnesium chelatase [Propionibacterium acnes]	326	0			
ATPase family associated with various cellular activities (AAA) [Propionibacterium acnes]	600	0			
hypothetical protein [Propionibacterium acnes]	88	9.02E-59			
hypothetical protein [Propionibacterium acnes]	48	1.16E-23	7	588	-
cobalamin biosynthesis protein CobN [Propionibacterium acnes]	95	3.86E-57			
cobalamin biosynthesis protein CobN [Propionibacterium acnes]	731	0	8	2,195	-
cobalamin biosynthesis protein CobN [Propionibacterium acnes]	166	5.33E-106	9	501	-
cobalamin biosynthesis protein CobN [Propionibacterium acnes]	283	0	10	2,762	-
iron ABC transporter permease [Propionibacterium]	269	0			
ferrichrome ABC transporter [Propionibacterium]	309	0			
sulfatase [Polaromonas sp. CG9_12]	39	$> 10^{-5}$			
ABC transporter substrate-binding protein [Propionibacterium acnes]	260	0	11	783	-
peptide ABC transporter ATPase [Propionibacterium humerusii]	94	6.22E-51	12	1,177	-
hypothetical protein [Propionibacterium]	248	7.74E-175	13	1,292	-
chitooligosaccharide deacetylase [Propionibacterium]	79	2.39E-48			
hypothetical protein [Propionibacterium acnes]	31	1.12E-12	14	1,754	KPA171202

flavin reductase [Propionibacterium acnes]<br>hypothetical protein<br>hypothetical protein [Propionibacterium]<br>hypothetical protein [Propionibacterium]<br>TetR family transcriptional regulator [Propionibacterium acnes]	185<br>15<br>95<br>72<br>56	1.88E-132<br>> 10^{-5}<br>1.33E-52<br>6.41E-43<br>6.96E-31			6609<br>HL030PA1<br>SK187
hypothetical protein<br>hypothetical protein [Propionibacterium acnes]	55<br>102	> 10^{-5}<br>4.87E-50	15	1,058	-
hypothetical protein<br>GNAT family acetyltransferase [Propionibacterium]<br>hypothetical protein [Propionibacterium]<br>hypothetical protein [Propionibacterium]<br>aminooxidase [Propionibacterium acnes]	51<br>82<br>243<br>97<br>424	> 10^{-5}<br>5.07E-53<br>0<br>5.64E-63<br>0	16	3,076	-
hypothetical protein<br>peptidyl-tRNA hydrolase [Propionibacterium]<br>multidrug transporter [Propionibacterium]	26<br>142<br>389	> 10^{-5}<br>9.95E-95<br>0	17	2,099	-
membrane protein [Propionibacterium]<br>conjugal transfer protein TraL [Propionibacterium acnes]<br>ABC transporter ATP-binding protein [Propionibacterium]	174<br>390<br>267	6.22E-117<br>0<br>0	18	3,156	-
ABC transporter substrate-binding protein_partial [Propionibacterium sp. 409-HC1]<br>maltose-binding protein [Propionibacterium]<br>maltose ABC transporter permease [Propionibacterium acnes]<br>hypothetical protein [Propionibacterium]<br>conjugal transfer protein TraL [Propionibacterium]<br>hypothetical protein [Dactylosporangium aurantiacum]<br>LacI family transcriptional regulator [Propionibacterium]	158<br>246<br>542<br>149<br>146<br>86<br>28	6.74E-53<br>1.36E-174<br>0<br>2.42E-102<br>1.27E-95<br>> 10^{-5}<br>2.42E-09	19	4,397	-
error-prone DNA polymerase [Propionibacterium acnes]<br>2OG-Fe(II) oxygenase [Propionibacterium acnes]<br>DNA repair nucleotidyltransferase [Propionibacterium acnes]	939<br>50<br>527	0<br>2.23E-27<br>0	20	4,718	-
hypothetical protein [Propionibacterium acnes]	214	2.57E-132	21	646	-
peptide ABC transporter substrate-binding protein [Propionibacterium acnes]<br>hypothetical protein<br>hypothetical protein [Propionibacterium]	90<br>25<br>28	3.04E-53<br>> 10^{-5}<br>6.31E-11	22	567	-
nuclease [Propionibacterium]<br>endonuclease VII [Propionibacterium]<br>iron ABC transporter ATP-binding protein_partial [Propionibacterium]<br>membrane protein [Propionibacterium]<br>ABC transporter ATP-binding protein [Propionibacterium]<br>hypothetical protein	582<br>87<br>508<br>761<br>221<br>15	0<br>9.77E-54<br>0<br>0<br>2.92E-154<br>> 10^{-5}	23	7,265	KPA171202<br>6609<br>HL030PA1<br>HL097PA1<br>PRP-38
sialic acid transporter [Propionibacterium]	534	0	24	1,756	-
zinc-binding dehydrogenase [Propionibacterium avidum]	220	1.27E-155	25	661	-
inosine-uridine preferring nucleoside hydrolase [Propionibacterium acnes]<br>hypothetical protein [Propionibacterium]<br>thioredoxin [Propionibacterium acnes]	141<br>149<br>53	4.63E-93<br>4.75E-92<br>1.17E-25	26	13,353	KPA171202<br>6609<br>HL030PA1

cupin [Propionibacterium]	158	9.91E-113			
TetR family transcriptional regulator [Propionibacterium]	214	2.88E-150			
ABC transporter [Propionibacterium]	597	0			
iron ABC transporter ATP-binding protein [Propionibacterium acnes]	606	0			
permease [Propionibacterium]	75	1.18E-34			
hypothetical protein [Propionibacterium]	227	3.15E-158			
membrane protein [Propionibacterium acnes]	423	0			
membrane protein [Propionibacterium]	234	6.53E-160			
ABC transporter permease [Propionibacterium acnes]	402	0			
ABC transporter ATP-binding protein [Propionibacterium]	244	1.53E-172			
diaminohydroxyphosphoribosylaminopyrimidine deaminase [Propionibacterium acnes]	355	0			
